# Urinary tract infection in adults: gaps in current guidelines – opinions from an international multidisciplinary panel and relevance to clinical practice

**DOI:** 10.1186/s12919-025-00333-5

**Published:** 2025-07-03

**Authors:** Kalpana Gupta, Florian Wagenlehner, Mark Wilcox, Sonali D. Advani, Manu Bilsen, Gernot Bonkat, Rafael Cantón, Suzanne Geerlings, Beatrice Grabein, Juan P. Horcajada, Pamela Kushner, Navaneeth Narayanan, Marc Scheetz

**Affiliations:** 1 Boston University School of Medicine, Boston, USA; 2 Veterans Affairs Boston Healthcare System, West Roxbury, USA; 3 Harvard Medical School, Boston, USA; 4 Clinic for Urology, Paediatric Urology and Andrology, Justus Liebig University, Giessen, Germany; 5 School of Medicine, Leeds Institute of Medical Research, University of Leeds & Leeds Teaching Hospitals, Leeds, UK; 6 ViiV Healthcare, Durham, USA; 7 Department of Infectious Diseases, Leiden University Medical Center, Leiden, Netherlands; 8 Alta Uro AG, Merian Iselin Klinik, Center of Biomechanics & Calorimetry, University of Basel, Basel, Switzerland; 9 Servicio de Microbiología, Hospital Universitario Ramón y Cajal, Instituto Ramón y Cajal de Investigación Sanitaria (IRYCIS), Madrid, Spain and CIBER de Investigación en Enfermedades Infecciosas (CIBERINFEC), Instituto de Salud Carlos III, Madrid, Spain; 10 Department of Internal Medicine, Amsterdam Institute for Infection and Immunity, Amsterdam Public Health Research Institute, Amsterdam, Netherlands; 11 Stabsstelle Klinische Mikrobiologie und Krankenhaushygiene, LMU Klinikum, Munich, Germany; 12 Infectious Diseases Service, Hospital del Mar; Hospital del Mar Research Institute (IMIM), Universitat Pompeu Fabra (UPF), Barcelona, Spain; 13 Centro de Investigación Biomédica en Red de Enfermedades Infecciosas (CIBERINFEC), Instituto Carlos III, Madrid, Spain; 14 Department of Family Medicine, University of California Irvine Medical Center, Orange, USA; 15 Department of Pharmacy Practice and Administration, Rutgers University Ernest Mario School of Pharmacy, Piscataway, USA; 16 Departments of Pharmacy Practice and Pharmacology, Colleges of Pharmacy and Graduate Studies, Midwestern University, Downers Grove, USA

**Keywords:** Antimicrobial de-escalation, Antimicrobial resistance, Complicated urinary tract infection, Guideline, Uncomplicated urinary tract infection

## Abstract

**Purpose:**

Although urinary tract infections (UTIs) are one of the most common infections encountered in clinical practice, many challenges remain with respect to classification and management. The purpose of this report is to discuss key issues in the management of UTIs and identify gaps in current knowledge and guidelines, as well as future research needs.

**Design:**

A multidisciplinary panel of 13 experts from 6 European countries and the United States met on April 27, 2024. They discussed predefined key clinical questions, including classification of UTIs, current management guidelines, management of UTIs in men, antimicrobial switching, and post-treatment asymptomatic bacteriuria.

**Results:**

The panel agreed that differentiation between complicated and uncomplicated UTIs is crucial to antimicrobial selection and can impact outcomes. In particular, definitions of complicated UTIs (cUTIs) vary widely between guidelines and in the literature. Patients with cUTIs are not a homogeneous group and differences in risk factors and prognosis should be considered. However, a balance must be sought between appropriate antimicrobial treatment and complexity of guidelines, which can hinder their implementation, especially in primary care. Guidelines published by the European Urology Association and the Infectious Diseases Society of America differ in their antimicrobial treatment recommendations for cUTIs, which is important at a time of increasing antimicrobial resistance. In men with UTIs, it has been established that a longer duration of antimicrobial therapy is needed in cases where fever is present. De-escalation from broad- to narrow-spectrum antimicrobials is recommended wherever possible, and is associated with similar outcomes in many patients relative to remaining on broad-spectrum treatment. Post-treatment asymptomatic bacteriuria should not be assessed, and treatment is not recommended. Non-specialist physician education is crucial to achieving better outcomes for patients with UTIs.

**Implications:**

Many challenges remain in the management of UTIs in adults, most notably making an accurate classification, which drives antimicrobial treatment selection. A balance between adequacy of management guidelines and their uptake in routine clinical practice is needed to improve outcomes.

## Introduction

Although urinary tract infections (UTIs) are one of the most frequently diagnosed and treated infections in clinical practice [1, 2], accurately classifying UTIs can be challenging. Furthermore, the distinction between ‘complicated’ and ‘uncomplicated’ UTIs, is ambiguous. Society guidelines typically apply the same definition of complicated UTI (cUTI) to different patient populations, although they may have varying underlying risks and complicating factors.

A multidisciplinary expert group from Europe and the United States (urology, infectious diseases, microbiology, primary care, and pharmacy) met for a workshop with the aim of addressing areas of clinical uncertainty in the classification and management of UTI according to current guidelines and published literature. Topics were selected by the steering committee, from their own clinical experience, and PubMed searches conducted in order to ascertain the quantity and quality of research findings available as a basis for highlighting gaps in the evidence base in each of these topics. Suggestions for further research are included in Table 1.

**Table 1:** Outstanding research and educational needs regarding classification and management of urinary tract infections in adults

Challenge	Research/educational needs
Current antimicrobial treatment	• High-quality studies of aminoglycoside monotherapy in patients with complicated UTIs and known resistance to other commonly used antimicrobials
Treatment of recurrent uncomplicated urinary tract infections in healthy young women	• Studies comparing different antimicrobials and/or longer duration of treatment
	• Reasons for non-response or recurrence after first-line antimicrobial treatment
	• Effectiveness of first-line antimicrobials in recurrent vs non-recurrent uncomplicated UTI
Patients with renal impairment	• Studies of aminoglycoside therapy (efficacy and safety)
	• Treatment success rates with standard vs higher doses/longer duration of treatment of first-line antimicrobials
Male patients	• Studies in men with cystitis only
Antimicrobial choice	• Development of a risk scoring system for AMR and poor outcomes
Antimicrobial de-escalation	• Large real-world studies of protocol-driven de-escalation and pre-defined clinical outcomes in specific patient populations
Post-episode asymptomatic bacteriuria	• Studies comparing clinical outcomes between patients with and without follow-up urine culture, especially in risk groups such as those with diabetes
Non-specialist understanding of UTI classification and appropriate treatment choice/guidance	• Case studies
	• Simple classification and treatment algorithm

*AMR* Antimicrobial resistance, *UTI*, Urinary tract infection

### Controversies in UTI classification

Accurately defining both uncomplicated UTI (uUTI) and cUTI is crucial to making appropriate diagnosis, which can result in substantially different management approaches [3]. uUTI is well-defined in both current European Association of Urology (EAU) and Infectious Diseases Society of America (IDSA) guidelines [1, 2]. (Revised IDSA guidelines are in development.) However, while in the EAU guidelines cUTI is typically patients that do not fit the uUTI criteria, an exception is made for uncomplicated pyelonephritis, which has a separate and distinct section. American Urological Association/Canadian Urological Association guidelines also classify UTIs with a multidrug resistant (MDR) organism and in immunocompromised patients as cUTIs [4]. In the published literature, definitions of cUTI vary greatly; in a review of 14 studies that defined cUTI, nine included both host factors and systemic involvement, indicating that definition of cUTI is problematic as different phenotypes are included in a single classification [5]. Additionally, both the United States Food and Drug Administration (FDA) and European Medicines Agency (EMA) have published recommendations concerning research in UTIs, which differ in symptom criteria and definitions [3, 6], while a more recent Delphi consensus panel has suggested new research standards in this area [7].

Some panel members no longer use the term ‘complicated’, instead preferring to base treatment decisions on severity of symptoms and the need for an antimicrobial with tissue penetration. This was also partly in response to primary care physicians who reported that guideline use of the terms ‘uncomplicated’ and ‘complicated’ was confusing and not relevant to clinical practice. One member cited the experience of certain groups of non-specialist physicians who might not differentiate between the different categories of UTI and then prescribe inappropriately, e.g., fluoroquinolones, or overtreat due to concerns regarding adequate antimicrobial coverage. In primary care the term ‘cystitis’ may be more useful. Although it might be useful in terms of future guidelines, avoiding the use of ‘complicated’ UTI has a number of issues, for example, the large number of studies including patients with cUTI and the ingrained nature of the term with healthcare professionals.

**Fig. 1:**
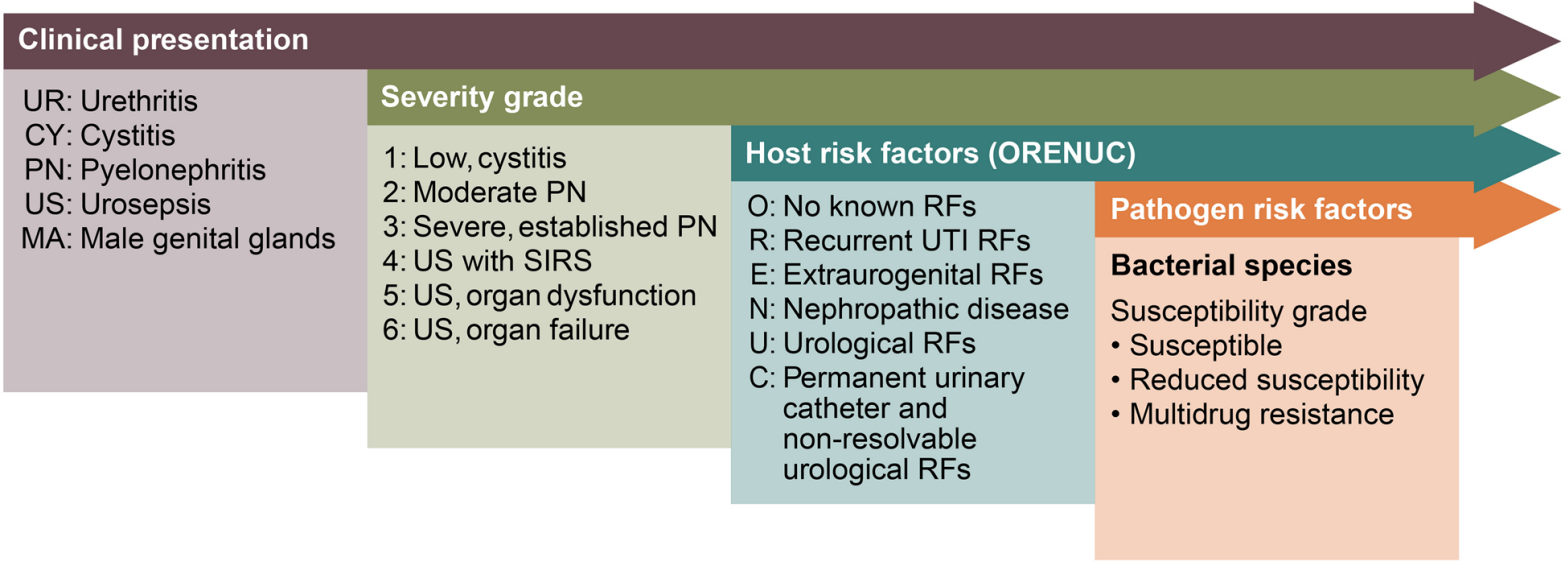
The European Section of Infections in Urology (ESIU) classification of urinary tract infections [8] RF, risk factor; SIRS, systemic inflammatory response syndrome. This figure has been reproduced with permission from Wagenlehner FME, et al. Nature Reviews in Urology 2020;17:586–600

Guidelines recommend different antimicrobial agents and regimens for the management of uUTI and cUTI, making an accurate distinction between them important (Table 2). These reflect that the bacterial spectrum is different in uUTI compared with cUTI, and the prevalence of AMR is higher in the latter [8]. The presence or absence of systemic symptoms and their severity are a key differentiator between uUTI and cUTI and these are included in the European Section of Infections in Urology classification, which provides specific categories of severity, alongside host and pathogen risk factors (Fig. 1) [8].

**Table 2:** Current European Association of Urology (EAU) and Infectious Diseases Society of America (IDSA) guidance for antimicrobial treatment of urinary tract infections

Uncomplicated urinary tract infection	
EAU (2024) [1]	• First-line: fosfomycin, nitrofurantoin, pivmecillinam
	• Alternatives: cephalosporins, e.g., cefadroxil, trimethoprim/TMP-SMX (if local resistance < 20%)
IDSA (2011) [2]	• First-line: fosfomycin, nitrofurantoin, pivmecillinam, TMP-SMX (if local resistance < 20%)
	• Alternatives: beta-lactams, fluoroquinolones (when recommended agents cannot be used)
**Complicated urinary tract infection**	
EAU (2024)	
Patients with systemic symptoms [1]	Intravenous (empirical): • Aminoglycoside ± amoxicillin • Second-generation cephalosporin + aminoglycoside • Third-generation cephalosporin • Extended-spectrum penicillin ± aminoglycoside
	• Treatment should be tailored according to local resistance data, urine culture/susceptibility testing results
	• Treatment should cover ESBLs if there is an increased likelihood of ESBL infection based on community prevalence, earlier urine cultures and prior antimicrobial exposure of the patient
Fluoroquinolones	Empirical ciprofloxacin only when local resistance is < 10% and: • Only when the entire treatment is given orally • Patients do not require hospitalisation • Patient has an anaphylaxis for beta-lactam antimicrobials/contraindications for third-generation cephalosporin or an aminoglycoside
	• Do not use fluoroquinolones for the empirical treatment of cUTI in patients from urology departments or when patients have used fluoroquinolones in the past 6 months
Recommendations against specific agent(s)	• Amoxicillin monotherapy, co-amoxiclav, trimethoprim and TMP-SMX unsuitable for empirical treatment due to high resistance rates
IDSA (2024) [9]	
Infections caused by ESBL-producing Enterobacterales	• Preferred: TMP-SMX, ciprofloxacin, levofloxacin (if susceptibility is demonstrated)
	• Ertapenem, meropenem and imipenem-cilastatin when resistance or toxicities limit use of TMP-SMX or fluoroquinolones
	• Alternative: aminoglycoside
Infections caused by CRE	• Preferred: TMP-SMX, ciprofloxacin, levofloxacin (if susceptibility is demonstrated), ceftazidime-avibactam, meropenem-vaborbactam, imipenem-cilastatin-relebactam, cefiderocol
	• Alternative: aminoglycoside

*cUTI* complicated urinary tract infection, *ESBL* extended-spectrum beta-lactamase, *TMP-SMX* trimethoprim-sulfamethoxazole

Specific risk factors have prognostic value for severe infections and treatment failure as these vary across different patient subgroups, indicating that they are not a homogeneous population (Table 3), although current guidelines do not take this into consideration. Many patients can fall into more than one subgroup, making management even more complex. The issue of age was discussed, but determination of ‘elderly’ patients is problematic and the panel suggested using frailty as a guide instead.

**Table 3:** Specific issues in patient subgroups with complicated urinary tract infections

Patient group	Specific considerations
Functional/anatomical abnormalities of the urinary tract	• Neurogenic bladder: may present with atypical symptoms, frequent catheterisation, high rates of resistant uropathogens [10, 11]
	• Vesicoureteral reflux: delayed antimicrobial treatment can be associated with increased risk of renal scarring [12]
Urinary catheterisation	• High antimicrobial treatment failure rates [13–15]
	• Causative bacterial species are different and polymicrobial UTIs are more frequent vs other cUTIs [16]
	• Increased risk of resistant uropathogens [14]
Renal stones	• Urinary obstruction can lead to ascending infections, bacteraemia, sepsis and death [17–19]
	• Broad-spectrum antimicrobials covering gram-negative and urease-producing organisms, e.g., *Proteus* spp., *Staphylococcus* spp. are typically required [20]
Diabetes mellitus	• Increased risk of resistant uropathogens [21]
	• More likely to be hospitalised with a UTI compared with patients without diabetes [21–24]
	• Bladder dysfunction secondary to diabetic neuropathy may be associated with atypical symptoms of UTI [25]
Renal transplant	• UTIs may be associated with decreased renal function and an increased risk of graft loss, especially with recurrent UTIs [26–28]
	• The presence of resistant uropathogens results in poorer transplant-related outcomes [29, 30]
Chronic kidney disease	• UTIs can be associated with ESRD, rapid eGFR decline and mortality [31]
	• Increased risk of hospital admission for UTI [22, 32]
	• Increased risk of urosepsis with decreasing eGFR [33]
	• Increased failure rates with standard antimicrobial therapy [34]
	• Need to consider dose adjustments of antimicrobials and nephrotoxicity [35]
Cancer	• Obstructive uropathy secondary to genitourinary solid tumours can lead to urosepsis and high levels of MDRO [36]
	• Risk of urosepsis and rehospitalisation following radical cystectomy with urinary diversion [37–39]
Autoimmune conditions	• MS: increased risk of UTI-related hospitalisation and mortality [40]
	• IBD: high frequency of urolithiasis, increased risk of UTI-related hospitalisation, rehospitalisation and acute kidney injury [41, 42]
	• RA: increased risk of UTI-related hospitalisations [43, 44]
	• Immunosuppressive therapy increases risk of UTIs [44, 45]
Pregnancy	• Increased risk of ascending infection [46]
	• Association with adverse maternal and foetal outcomes [46–48]

*cUTI* complicated urinary tract infection, *eGFR* estimated glomerular filtration rate, *ESRD* end-stage renal disease, *MDRO* multidrug resistant organisms, *UTI* urinary tract infection

Panel members felt that upper versus lower tract UTI is also an important distinction that drives antimicrobial treatment selection. In uUTIs, drugs that accumulate solely in the urine in therapeutic concentrations can be considered whereas this is not appropriate for more extensive systemic infections. The requirement for tissue penetration of an antimicrobial is especially important when considering infections such as prostatitis [49]. Notably, although uncomplicated pyelonephritis and cUTI are classified separately in the EAU and IDSA guidelines, some of the panel felt that the former may be considered as a cUTI in clinical practice due to the presence of systemic symptoms [1, 9].

There were differences among the group regarding the management approach for recurrent episodes of cystitis in younger healthy women, namely whether these episodes should be always be managed as a discrete uUTI (based on symptoms alone) or whether they should be considered as a cUTI. This classification has implications regarding the choice of treatment for each subsequent episode, specifically, whether different antimicrobials and/or a longer duration of treatment are required. Current published guidance recommends treatment with first-line antimicrobials for an individual episode of uUTI (after review of prior urine cultures and allergies), unless antimicrobial resistance (AMR) is suspected [4]. This is particularly relevant as it has been shown that resistance to trimethoprim-sulfamethoxazole (TMP-SMX) and fluoroquinolones is greater in recurrent uUTI than in non-recurrent uUTI [50]; anecdotally, this is not the case for other first-line treatment options but clinical study evidence is lacking. Possible additional underlying reasons for treatment non-response or recurrence should be investigated further. The group also questioned whether all postmenopausal women with an apparent uUTI should automatically be categorised as cUTI.

### Current guidance for antimicrobial treatment

Current guidance for selection of antimicrobial treatment in uUTI is similar in the EAU and IDSA guidelines but differs in the management of cUTI; the former also includes specific recommendations regarding antimicrobial stewardship while the latter has a specific focus on AMR infections [1, 9] (Table 1).

When considering the guidelines versus real-life clinical practice, aminoglycoside monotherapy is recommended for the treatment of UTIs, but the majority of the panel agreed that they do not prescribe this routinely. This approach reflects the lack of published evidence, as the majority of studies have evaluated aminoglycosides in combination with cephalosporins. However, a meta-analysis published in 2007 indicated non-inferiority of aminoglycoside monotherapy compared with standard antimicrobial therapy in UTIs [51], and one more recent randomised controlled trial demonstrated non-inferiority of plazomicin monotherapy to meropenem in patients with cUTI (including acute pyelonephritis) [52]. One expert stated that they use aminoglycoside monotherapy in situations where multidrug resistant *Pseudomonas* is a concern, and another indicated that in their centre a protocol is under development for a single dose of aminoglycoside in situations where there is an issue regarding resistance to most antimicrobials commonly used for UTIs. A key unknown is whether aminoglycosides can be used safely in patients with renal impairment.

Furthermore, aminoglycoside monotherapy may also have a place in UTIs where no AMR has been detected, in order to reduce the selective pressures associated with other antimicrobials. Overall, there is limited evidence to support these approaches and more research is needed. The low real-world usage of aminoglycosides is likely related to safety concerns where other options are generally regarded as safer (especially with respect to associated organ toxicity). Further clinical research is required to determine if clinicians’ overall perspectives regarding aminoglycosides are supported by the evidence.

In general, there are several barriers to the use of carbapenems and old and new beta-lactam/beta-lactamase inhibitors (BL/BLIs) for the treatment of cUTI across countries and individual centres. Firstly, the current EAU guidelines do not specify these classes of antimicrobials in their recommendations for the treatment of non-septic cUTI, although general statements refer to the need for extended-spectrum beta-lactamase (ESBL) coverage [1]. In contrast, the IDSA guidelines explicitly recommend novel BL/BLIs as an option for cUTIs caused by carbapenem-resistant Enterobacterales (CRE) (ceftazidime/avibactam, meropenem/vaborbactam, and imipenem/cilastatin/relebactam) [9]. Secondly, the majority of the panel agreed that formularies and antimicrobial stewardship committees typically require evidence of resistance to other antimicrobials (or susceptibility profile of the organism) prior to their prescription of these agents. However, in one centre meropenem alone is often used for patients with renal impairment and another agreed that meropenem is more easily available than the novel BL/BLI combinations. Such restriction is likely due to their broad-spectrum activity, and is generally in line with the World Health Organization AwaRE Reserve category of antimicrobials with respect to BL/BLIs [53]. In the United States there is an effort to report use of certain antimicrobials to the CDC as part of a national healthcare surveillance network. Cost and lack of clinical experience are also major factors with respect to the newer antimicrobials in many centres. The panel agreed that the lists published in guidelines do not typically indicate a sequence in which specific antimicrobials should be used.

A key contrast between the EAU and IDSA guidelines is the recommendation for the use of TMP-SMX and fluoroquinolones. While the EAU guidelines recommend against TMP-SMX due to high resistance rates and restrict fluoroquinolones to cases where AMR is < 10%, the IDSA guidelines list these agents among the preferred antimicrobials for patients with cUTIs caused by ESBL producers and CRE, although confirmation of susceptibility is required [1, 9]. These agents have excellent urinary activity and high efficacy if the uropathogen is susceptible. Although resistance rates of common uropathogens to both TMP-SMX and fluoroquinolones are high in both Europe and North America [54, 55] and the prevalence of ESBL producers is also increasing [56, 57], they are not uniform or absolute. Thus, an active assessment for risk of resistance is needed before empiric use and culture confirmation should be done. Moreover, the panel agreed that simultaneous resistance to TMP-SMX and fluoroquinolones in UTIs is typical in clinical practice, especially in ESBL producers and CRE, which is supported by the literature [58, 59].

The panel felt that although the EAU guidelines state that empirical treatment must be based on ‘local’ surveillance data, AMR profiles may differ within countries and even hospitals in the same region [1]. Additionally, hospital antibiograms typically reflect more severe UTIs and are not representative of uUTI. Many of the panel are members of guidelines committees but stated that they adapt guidelines for local use, taking such factors into account. Perhaps more importantly than considerations of resistance to fluoroquinolones, the adverse effects of these antimicrobials and the safety warnings issued by the FDA and EMA must be borne in mind when being used for uUTI [60, 61].

Individuals with renal impairment/chronic kidney disease comprise an important proportion of patients with UTIs [22, 32], but there is limited guidance on antimicrobial regimens regarding this group. However, it has been suggested that failure rates with standard therapy are increased in such patients [34]. One panel member suggested that there are several factors that can guide treatment decisions in such patients, including age, degree of renal impairment, and antimicrobial availability. Another panel member proposed that patients with renal impairment should receive lower doses of antimicrobials but for a longer duration of treatment. In contrast, a third suggested that treatment failure in patients with severe UTIs and renal impairment may be related to the dose being insufficient. The panel agreed that this is an important area for further research.

### Urinary tract infections in men

It is well-established that UTIs in men are less common than in women (with perhaps the exception of catheter-related UTIs and in institutionalised populations), with a ratio of approximately 1:4 [54, 62]. Additionally, the bacterial makeup differs between the two sexes, with a lower proportion of UTIs caused by *Escherichia coli* and higher proportion of *Pseudomonas aeruginosa* in men relative to women [54, 63]. In addition, primary care physicians may feel less confident diagnosing and managing UTIs in men [64].

Men have traditionally been considered to have cUTIs [1], although in a review of primary care guidelines, terminology differs between countries (cUTI, cystitis, prostatitis, general male UTI), along with treatment recommendations [65]. Notably, the majority of men, especially in primary care, present with symptoms suggestive of cystitis (although this could also be prostatitis based on symptoms alone) [66, 67], and one panel member questioned why such patients could not be treated as per uUTI. The EAU guidelines classify UTIs in men as cUTI, but there is a contradiction as they do make a separate recommendation of 7 d treatment with TMP-SMX in men with uUTI (compared with either TMP-SMX or a fluoroquinolone for at least 7 d in cUTI) [1].

Studies have shown that systemic symptoms such as fever should be a key differentiator for length of antimicrobial therapy in men. In one randomised study, treatment for 7 d with TMP-SMX or ciprofloxacin was non-inferior to 14 d with the same treatment of UTIs in afebrile men [68]. Conversely, in two studies of febrile men with UTIs, 7 d of antimicrobial treatment was statistically inferior to 14 d of the same treatment regimen (even though in one of the studies the clinical success rates were comparable) [69, 70]. The optimal duration of therapy for febrile UTI in men therefore remains unclear; while some of the panel treat patients with systemic symptoms with 14 d of antimicrobial therapy, others felt that a shorter course of treatment might be sufficient if the patient responds quickly. Another key differentiator is whether the UTI is community- or hospital-acquired, or healthcare-associated, as these are typically caused by different organisms. Men also represent a heterogeneous population, e.g., with respect to involvement of the prostate. Notably, in primary care studies, reported rates of treatment failure in men with cystitis (the need for further antibiotic prescriptions or worsening symptoms) with nitrofurantoin, fosfomycin, pivmecillinam and TMP-SMX were ~ 25%, 22%, 25% and 14–22%, respectively; but marginally lower for fluoroquinolones (10–19%) [67, 71]. Treatment failure rate increased with age for all agents.

### Switching between narrow- and broad-spectrum antimicrobials

The panel agreed that identification of patients at increased risk of AMR/MDR is important when considering antimicrobial treatment options for UTIs (Table 4). However, although individual risk factors may not have high predictive capability, the presence of multiple risk factors increases likelihood of AMR. A few of the panel suggested a risk scoring system, with weighting of the individual factors, which has been suggested previously [55]. Severity of illness is often a primary driver behind antimicrobial selection, and an adequate assessment is therefore crucial. If a patient’s condition worsens during the period when waiting for urine culture results, then initiation of a broad-spectrum antimicrobial was considered by the panel as a reasonable approach. Conversely, if a patient’s condition improves rapidly (typically within 72 h), de-escalation of treatment might be an option. Escalation from a narrow- to broad-spectrum antimicrobial may also be necessary once urine culture results are available.

**Table 4:** Risk factors for antimicrobial/multidrug resistance in urinary tract infections

Age > 50 years [72, 73]
Recent hospital admission [74]
Long-term residential care [75]
Healthcare contacts within the previous 3–6 months [74, 76]
Urinary catheters [72]
Urogenital abnormalities [72, 77]
Prior infection with resistant organisms [73, 75, 78]
Chronic conditions (diabetes mellitus, hypertension, chronic kidney disease) [21, 78, 79]
Prior antibiotic use within 90 days [73, 75–78]
Recurrent urinary tract infection [77]
Travel to areas with a high prevalence of antimicrobial resistance [72, 76]

The concept of antimicrobial de-escalation is complex and different studies have employed varying definitions. Many have used categories of antimicrobial spectrum, from narrow-, through broad- to extended-spectrum (and protected, as per WHO AwaRE guidance [53]), with the ultimate aim of switching to narrow-spectrum therapy according to urine culture results [80, 81]. Alternatively, others have considered a switch from carbapenems to any other appropriate alternative antimicrobials as de-escalation [82]. De-escalation may also include the switch from intravenous (IV) to oral therapy, and a shorter duration of treatment [81, 83].

There are a number of studies reporting the frequency of de-escalation in clinical practice for the management of cUTIs, and the majority have shown no detriment to clinical outcomes (Table 5). Notably, in some of these studies the majority of patients meeting de-escalation criteria were not switched. Ultimately, the bioavailability of the active compound and amount that reaches the target site is the most important factor and not the route of initial administration. The panel felt that clinicians should make treatment change decisions on this basis and with the idea that oral therapy can require less complex care because of ease of administration and monitoring as well as fewer infection/contamination risks with intravascular devices.

**Table 5:** Studies reporting antimicrobial therapy de-escalation and outcomes in patients with urinary tract infections

	Study population	De-escalation criteria	Patients meeting criteria for antimicrobial de-escalation	Patients who received de-escalation of treatment	Reported outcomes associated with de-escalation
Wells 2023 [84]	Hospitalised patients with cystitis, pyelonephritis or unspecified UTI (n = 300)	Switch from IV ceftriaxone to oral antimicrobial during admission according to pharmacist criteria	264 (88%) met criteria for de-escalation	36 (14%) during hospitalisation and 176 (67%) on discharge	None reported
Spoorenberg 2014 [85]	Hospitalised patients with cUTI (n = 1252)	Switch from IV to oral antimicrobial at 72 h according to safety criteria	543 (43%) met criteria for de-escalation	295 (54%)	Reduced length of stay
Gamble 2022 [86]	Hospitalised patients with non-bacteraemic UTIs caused by ESBL-producing pathogens (n = 153)	Switch from IV carbapenem to an oral antimicrobial within 120 h of carbapenem initiation according to clinical judgement and urine culture results	Not applicable	58 patients (38%) switched to oral antimicrobial therapy	No difference between continued IV carbapenem and oral antimicrobial therapy for recurrent UTI, readmission and mortality
Alshareef 2020 [83]	Hospitalised patients with UTI (n = 91)	Switch from IV broad-spectrum antimicrobial to culture-directed IV or oral narrow-spectrum antimicrobial	Not applicable	27 patients (30%) received antimicrobial de-escalation	Presence of MDR pathogens associated with broad- to narrow-spectrum treatment de-escalation failure

*cUTI* complicated urinary tract infection, *ESBL* extended-spectrum beta-lactamase, *IV* intravenous, *MDR* multidrug resistant, *UTI* urinary tract infection

### Post-treatment asymptomatic bacteriuria

The key data for discussion of whether asymptomatic bacteriuria should be assessed and treated following symptom resolution/improvements in UTIs came from an analysis of data from 13 Phase 3 clinical trials that were submitted to the US FDA [87]. Results of this analysis found that there was a five-fold increase of clinical relapse in patients without microbiological eradication at the end of treatment (although indeterminate outcomes were classified as relapse, potentially impacting the outcome). However, a few members of the panel indicated that the methodology of this analysis was flawed, making interpretation of the results difficult. In routine clinical practice, follow-up urine culture for assessment of microbiological eradication following treatment, i.e., presence of asymptomatic bacteriuria is not typically requested. Recurrence of UTIs is considered clinically according to symptoms rather than microbiological failure; patients will return if their symptoms re-emerge. It is unclear whether patients with risk factors (e.g., older age, diabetes) should have a repeat urine culture and more studies in this area are needed.

The greatest issue is the prevalence of asymptomatic bacteriuria overtreatment due to lack of understanding of this situation and documentation [88, 89]. This is especially relevant in older women residing in long-term care facilities. Studies have shown that antimicrobial therapy in patients with asymptomatic bacteriuria does not reduce the likelihood of UTIs compared with no treatment (and may even be associated with an increased risk) [90, 91]. In addition, treatment may be associated with an increased frequency of adverse events and is also an important driver of AMR that could impact future treatment of symptomatic UTIs. With some classes of antimicrobials, e.g., fluoroquinolones there is a risk of dysbiosis and its sequelae, including secondary infections. Current guidelines therefore recommend treatment of asymptomatic bacteriuria only in specific patient groups, e.g., pregnant women [1, 89].

### Clinical guidelines uptake and impact

There are numerous influences on implementation of guideline recommendations for UTIs, including clinical decision support systems, knowledge and training, peer-to-peer interactions, formularies/antimicrobial stewardship and length of guidelines [92, 93]. Consequently, adherence to guidelines for the management of UTIs remains variable [94]. The greatest issue is inappropriate prescribing of antibiotics, e.g., fluoroquinolones, and also overtreatment.

The group agreed that a simple visual representation of guidance/an algorithm, especially for non-specialists/primary care physicians would be useful, e.g., one previously published for diabetes [95]. This could be disseminated easily by multiple channels, including social media, and users could access further information supporting the algorithm via a journal publication. Case studies with feedback are also an important educational tool for primary care physicians.

## Summary

UTIs, specifically cUTIs, are complex and there are several unmet needs. Accurate distinction between uUTI and cUTI is the greatest issue when considering disease management, specifically choice of antimicrobial agent and duration of treatment. Overall, identification and classification needs to be simplified, and current guidelines are too complex, especially with respect to the interpretation in primary care. It must be remembered that patients with cUTI are not a homogeneous population and a key gap in current guidelines is a treatment approach for specific subgroups based on their risk of severe and systemic infections, comorbidities, and physiological changes that can impact drug pharmacokinetics and pharmacodynamics.

UTIs in men are generally considered as cUTI, although in many circumstances presentation suggests cystitis. The optimal duration of antimicrobial therapy in men with and without systemic symptoms (particularly fever) is unclear.

There is little evidence to support the empirical use of carbapenems and BL/BLIs in UTIs. AMR is increasing, limiting the use of some guideline-recommended antimicrobials, although resistance prevalence is highly variable between and within geographic regions. A number of risk factors for AMR/MDR have been identified, which could influence treatment selection. The concept of antimicrobial de-escalation is complex and not included in guidelines, although studies suggest it is achievable in some patients and is not associated with poorer outcomes.

Asymptomatic bacteriuria should not be investigated following clinical resolution of UTIs and treatment is recommended in only pregnant women and patients undergoing invasive urological procedures.

Finally, adherence to UTI management guidelines remains suboptimal, and reducing the unnecessary and inappropriate use of broad-spectrum antimicrobials remains essential.
